# Antibiotic Misuse in Wound Care: Can Bacterial Localization through Fluorescence Imaging Help?

**DOI:** 10.3390/diagnostics12123207

**Published:** 2022-12-17

**Authors:** Wayne J. Caputo, Patricia Monterosa, Donald Beggs

**Affiliations:** 1 Director of the Wound Care Center at Clara Maass Medical Center, Belleville, NJ 07109, USA; 2 Clara Maass Medical Center, Belleville, NJ 07109, USA; 3 Infectious Disease, Clara Maass Medical Center, Belleville, NJ 07109, USA

**Keywords:** antibiotic, antibiotic resistance, antibiotic stewardship, autofluorescence imaging, fluorescence imaging, bacterial count, inappropriate prescribing, medical overuse, over prescribing, wound healing

## Abstract

(1) Background: Systemic antibiotic use in chronic wounds is alarmingly high worldwide. Between 53% to 71% of patients are prescribed at least one course per chronic wound. Systemic antibiotic use should follow antibiotic stewardship guidelines and ought to be reserved for situations where their use is deemed supported by clinical indications. Unfortunately, in the field of wound care, indiscriminate and often inadequate use of systemic antibiotics is leading to both patient complications and worsening antibiotic resistance rates. Implementing novel tools that help clinicians prevent misuse or objectively determine the true need for systemic antibiotics is essential to reduce prescribing rates. (2) Methods: We present a compendium of available systemic antibiotic prescription rates in chronic wounds. The impact of various strategies used to improve these rates, as well as preliminary data on the impact of implementing fluorescence imaging technology to finesse wound status diagnosis, are presented. (3) Results: Interventions including feedback from wound care surveillance and treatment data registries as well as better diagnostic strategies can ameliorate antibiotic misuse. (4) Conclusions: Interventions that mitigate unnecessary antibiotic use are needed. Effective strategies include those that raise awareness of antibiotic overprescribing and those that enhance diagnosis of infection, such as fluorescence imaging.

## 1. Introduction

### Rationale

Antibiotics have changed the course of medicine. Eighty years since the advent of the antibiotic era, it is evident that the world, including the field of chronic wound care, would be very different without these life-saving drugs. Unfortunately, indiscriminate, and sometimes inappropriate antibiotic use has resulted in high rates of antibiotic resistance. In recent years, the Centers for Disease Control and Prevention (CDC) reported that approximately 30% of antibiotics are prescribed needlessly [1,2].

While the mainstay treatment of chronic, hard-to-heal wounds is to address the underlying cause (e.g., improve blood flow, avoid constant compression, improve glycemic levels, etc.), bacterial presence is a central part of the problem. Besides escalating the inherent risk of infection, bacterial growth above 10^4^ CFU/g, polymicrobial-type colonization, and biofilm, have all been linked to delayed healing, sepsis, and other increasing factors on morbidity and mortality [3]. The potential negative impact of bacteria in wounds has led wound care specialists to the regular use of antimicrobials, including systemic antibiotics, as part of the treatment of hard-to-heal wounds [4,5]. This is often despite unclear indications for their use. Most bacterial presence in chronic wounds can and should be addressed locally and by physical removal [6]. However, it is challenging to determine clinically where and when to do so safely and effectively and when that further step for systemic antibiotics needs to be made. Clinical signs and symptoms are often unreliable markers of high bacterial presence, biofilm, and infection [7,8]. This rings particularly true in the generally immunocompromised chronic wound patient population (e.g., diabetic patients, elderly). These hard-to-diagnose patients are also at higher risk of severe complications such as sepsis [9]. Diagnostic uncertainty, added to a higher risk of poor evolution, can lead to haphazard and erroneous empiric antibiotic prescribing, a phenomenon all too prevalent in chronic wound care [10,11]. Outpatient chronic wound patients receive significantly more antibiotic prescriptions than age and gender-matched patients without wounds [12], and prescribing rates in the inpatient and long-term care settings are even higher [13].

Over prescription of antibiotics leads to unfortunate consequences for patients such as toxicity and *C. difficile* infections, amongst others [14]. On a larger scale, over-prescription and antibiotic misuse heavily contribute to increasing rates of antibiotic resistance. Despite strong suggestion that the field of wound care has taken a haphazard approach to prescribing, this has not been well documented in the literature. The present article aims to collect the recent, available information on rates of systemic antibiotic prescription, specifically for the field of chronic wound care.

Secondarily, we will discuss and report on strategies employed globally to combat antibiotic overuse and misuse, some designed to fight antibiotic resistance. Some strategies focus on raising awareness and assessment of self-reported antibiotic usage, while others focus on enhancing the diagnostic process of bacterial detection and localization to better support (or prevent) prescription of systemic antibiotics.

Next, we will present our experience with one particularly innovative strategy, in the form of a point of care fluorescence (FL) imaging device. The implementation of this device in our daily clinical evaluations has improved the bacterial-infection management in our patients. This approach has facilitated improved local treatment and has enriched the decision-making process behind systemic antibiotic prescribing.

## 2. Materials and Methods

A review of the literature was conducted for studies reporting systemic antibiotic prescribing for chronic wound care and/or wound healing with or without strategies for antibiotic use optimization.

**Search strategy**: The literature review was conducted on 1–15 July 2022 across Pubmed for publications in English from 1 January 1990 to 30 June 2022. Articles published prior to 1990 were not included because: (1) they would not contain recent information on prescription rates, thus, are less relevant to today’s practitioners (2) There is no clear focus on strategies for rational systemic antibiotic use and prescription. Literature published before 1990 would pre-date modern antibiotic stewardship programs, the CDC’s National Antimicrobial Resistance Monitoring System for Enteric Bacteria (NARMS), and in general the global awareness of this threat as it exists at present. The search strategy incorporated specific search terms including ‘chronic wounds’, ‘systemic antibiotic overuse’, ‘antibiotic stewardship’, ‘diagnostic stewardship’ and ‘systemic antibiotics’. Boolean operators were employed when needed (e.g., ‘antibiotic overuse’ “AND” ‘chronic wounds’). This was complemented by a hand search of some wound care journals (e.g., *Advances in Wound Care*, *Diagnostics*, *Wound Repair and Regeneration*).

***Inclusion criteria:*** (1) Reported systemic antibiotic prescription rates based on standard of care; (2) rates reported were specific to chronic wound care (3); if any strategies of antibiotic use optimization were employed, they were noted and their impact on antibiotic prescription rates was recorded (however, the absence of these data was not considered a reason for exclusion).

***Preliminaryreport on our clinical study:*** We report our experience with a fluorescence imaging device by conducting a retrospective, observational evaluation of the impact of FL-imaging over a one-year period (October 2020 to October 2021) on diabetic foot ulcer (DFU) and venous stasis ulcer (VLU) patients that had been imaged during their care at the outpatient Wound Care Center at Clara Maass Hospital in New Jersey, USA. FL- imaging was performed using a point-of care imaging device (MolecuLight^®^) that can detect bacterial loads >10^4^ CFU/g. High bacterial loads are often pathogenic [4,5,15] and indicate the need for clinical intervention. This device has been extensively validated for its ability to detect high bacterial loads [8,11,16,17,18,19,20] and has been used in other studies to inform wound care treatment plans [16,17,21].

We established a series of parameters that guided our clinical approach to FL-imaging and wound care treatment strategies, depicted in the following ***workflow:***

**Figure 1:**
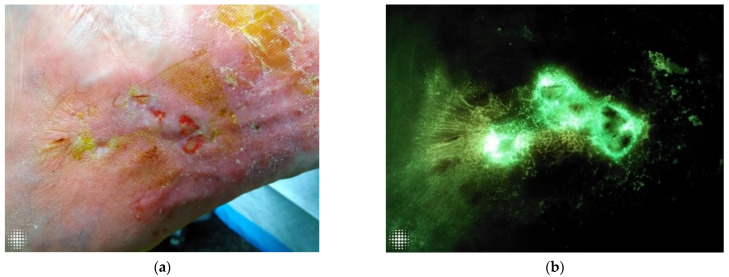
(**a**). A visually innocuous wound on clinical inspection. (**b**) The same wound was assessed under FL-imaging, demonstrating cyan positive signal, indicative of *Pseudomonas*. This was later confirmed by microbiology.

1. Any chronic wound with prior history of infection, any current signs or symptoms of infection, or exhibiting delayed healing was imaged for regions containing high bacterial loads.
2. A wound with a positive FL-image (>10^4^ CFU/g bacterial load), as indicated by a red or cyan signal (Figure 1), was addressed at the locations indicated by images through a combination of sharp or blunt debridement and cleaning.
3. After these hygiene-based procedures, the wound was re-imaged to determine the effectiveness of those strategies and our approach was revised when needed to include more aggressive hygiene (e.g., higher-level debridement) or hygiene over a larger area.
4. If a cellular tissue product (CTP) was to be utilized, the wound bed had to display a negative FL- image before placement took place. If there were more than one possible location for the CTP, best location was determined based on FL-image findings.
5. If re-imaging after more aggressive hygiene revealed that the fluorescence bacterial signal was still not fully eradicated, we implemented the use of topical antimicrobials (ointments and/or dressings).
6. As a last resort, when topical strategies could not address the bacterial signal, or in patients with evident and persistent clinical evidence of infection despite the implementation of all other strategies, oral antibiotics were prescribed.

## 3. Results

***Antibiotic prescription rates in the chronic wound population:*** Reports of systemic antibiotic prescribing rates in chronic wound outpatients are lacking. Most described outpatient settings and there was little to no information on inpatient prescribing. Overall, we found six publications that met the inclusion criteria and reported on antibiotic prescription rates in the chronic wound population. Half of these described the interventions employed to optimize antibiotic use and two reported on post-interventional systemic antibiotic prescription rates. Findings for outpatient wound care antibiotic prescribing are listed in Table 1. Antibiotic prescribing rates were *very high* amongst wound care patients, with 53.3% to 71% of patients being prescribed a wound-related antibiotic at some point during their outpatient wound care [7,16,22,23,24].

***Preliminary report on clinical study:*** The retrospective analysis of our patient population included a consecutive 69-patient cohort in which FL-imaging had been performed during at least one of their patient visits (typically at 3 or more, until bacterial burden was under control). Each patient had a custom treatment plan in place that was designed to consider FL-imaging findings to directly address their bacterial-infection challenges. We created these patient-specific plans using a procedure similar to that of consensus guidelines on implementing this technology from Oropallo et al. [18].

All of the patients in this 69-patient cohort were treated with local, targeted measures that included serial wound hygiene, debridement and topical antibiotics, per standard of care. However, by utilizing an imaging-informed approach (i.e., customized treatment plans that include FL-imaging to inform clinical decision-making), hygiene-based decisions (debridement, cleansing) were impacted in 70% of wound visits and our antimicrobial prescribing decisions changed at the bedside in 41% of the visits. Using the workflow outlined in the ***results*** section above for wound-specific decision making around bacterial presence, only 3/69 patients (4.3%) required the use of systemic antibiotics at any point during their wound care at our facility.

## 4. Discussion

Soft tissue and skin infections (SSTIs), including chronic, non-healing wounds are a very frequent outpatient and inpatient ailment. A recent study in the US looked at 2.3 million SSTIs diagnoses and found their occurrence to be twice that of UTIs [25]. Many of these SSTIs are treated with systemic antibiotics. According to a report published by the Journal of the American Medical Association (JAMA) and the Center for Disease Control and prevention (CDC) at least 1 in 3 antibiotic prescriptions are unnecessary [2]. A retrospective cohort study by Hurley et al. [26] assessed the frequency of antibiotic exposure and found that nearly half of all uncomplicated SSTIs in ambulatory care settings receive avoidable antibiotics. This kind of practice has contributed to the alarming antibiotic resistance crisis. According to the World Health Organization, we now find ourselves in a situation where there are not enough antibiotics in development to be able to fight multi-drug resistant infections [27].

Certain settings and types of infections are more likely to receive antibiotics. In settings where patients are more vulnerable, tend to decompensate rapidly, and are at a greater risk of complications, antibiotic use may be more frequent. The same is true in cases of torpidly evolving pathologies where the infectious culprit is not easily identifiable. Chronic wounds and the patients that typically suffer from them quite often share all these characteristics. Non-healing wounds are considered complex, polymicrobial, and plagued with high loads of bacteria, leading these patients to regularly receive systemic antibiotics [5,28,29,30,31]. A prime example of typical and complex chronic wound care patients are the residents of long-term care facilities and other prolonged inpatient settings where antibiotic use is hardly regulated. In fact, a recent survey demonstrated that 79% of residents in long term care facilities received at least one course of systemic antibiotics per year [32] and evidence has also shown that between 25–75% of the indications for those prescriptions have been inadequate [33]. There is little data available on the proportion of those prescriptions which are attributed to SSTIs, including SSTIs in chronic wounds.

### 4.1. How Profound Is the Systemic Antibiotic Prescription Rate Issue in Wound Care?

Systemic antibiotic prescribing rates are sparsely available in the literature for the chronic wound patient population. The series identified in the present review, which include rates from multiple sites across Europe and North America, demonstrate alarmingly high prescription rates, up to 71% per wound’s lifetime; moreover, rates that substantially greater than those encountered in the non-chronic wound population. A study by Howell-Jones et al. reports an average of 3.39 antibiotic courses in their chronic wound study population compared to 2.15 antibiotic courses in the non-chronic wound population (*p* < 0.001) [22]. Similarly, Gürgen et al. found that 25% of the patients who had received antibiotics in their study cohort had two or more antibiotic courses prior to their arrival at the wound healing unit. For the most part, these were administered in cases of non-healing post-surgical wounds and venous and arterial leg ulcers. These authors re-evaluated the need for previously received systemic antibiotics in a cohort of 105 patients and concluded that previously received systemic treatment was pertinent in only 1/105 patients, while 6/105 of the patients that did not receive this treatment indeed should have [7]. Howell-Jones et al. found that chronic wound patients were prescribed on average more broad-spectrum antibiotics than patients with no chronic wounds [22]. A commonly found poor practice is that antibiotic prescriptions are often based on expert opinions rather than scientific facts. A study by Serena et al. found that antimicrobials (including dressings, topicals, and systemic antibiotics) were prescribed at a similar rate for wounds identified as CSS+ (75.0%) and CSS− (72.8%, *p*  =  0.76) and a third of those that were prescribed antibiotics did not exhibit any CSS of infection [11].

Other studies have shown that clinical signs and symptoms are not routinely used to determine the need for systemic antibiotics, as is suggested by guidelines [34,35]. In some instances, this may stem from the lack of reliability and muted expression of the signs and symptoms themselves. A clinical trial by Le et al. conducted across 14 US wound care centers demonstrated this through the use of FL-imaging to identify high bacterial loads in chronic wounds; clinical signs and symptoms did not correlate to the levels of bacterial presence in 85% of the wounds [8]. In other instances, system-dependent factors may be at play. A retrospective analysis by Yogo et al. undertaken in long term care facilities across the Denver metropolitan area showed that over 50% of the antibiotics used for suspected skin infections, including cellulitis and infected wound/ulcers, were prescribed over the phone, and that 41% of those patients had no physical exam follow-up by a health care provider within the 48 h after the course was initiated [36]. Other factors may be inherent to the unique characteristics of patients in the chronic wound realm. Lipsky et al. argues that haphazard approaches to systemic antibiotic prescription may originate from a number of reasons, including diagnostic uncertainty paired with fear of a poor outcome or complications in a particularly frail population. There may also be pressure for antibiotic prescription placed on the clinician by the patients themselves [10]. Perhaps some of the confusion stems from lack of conclusive evidence to either support or oppose the use of systemic antibiotics in chronic wounds [37]. Therefore, guidelines cannot present definitive and conclusive recommendations other than supporting this decision in testing that delays the initiation or termination of the antibiotic [34,35].

Considering irregular prescribing and treatment practices, some strategies are being implemented. These are based on the monitoring and evaluation of he provided to chronic wound patients. Detrimental, inefficient and unsupported practices are flagged, and this then leads to their modification and the optimization of care. This was demonstrated by Öien et al. [24,38], where a National Quality Assessment Registry for Ulcer Care was implemented and studied, thus providing a control mechanism for health care providers, as well as a source of feedback on the potential areas for therapeutic optimization. Its impact was noticeable with a 53% decrease on systemic antibiotic consumption and a shorter time to heal for chronic ulcers, 3 years after it implementation: the median healing time for all ulcers decreased significantly from 146 days in 2009 to 63 days in 2012, and for venous ulcers specifically, from 120 days in 2009 to 69 days in 2012. This was accomplished while systemic antibiotic use was reduced by 63% [24]. At present, initiatives to monitor and evaluate the management of ulcers has been undertaken by Germany, Australia, and the European Wound Management Association (EWMA) [38,39]. In the United States, the Joint Commission has mandated that wound care providers have an antimicrobial stewardship plan in place, and others suggest that tools based on clinician and patient education could improve antibiotic stewardship [10].

### 4.2. MolecuLight Fluorescence (FL) Imaging of High Bacterial Load and Our Clinical Experience

In essence, strategies aimed to optimize the treatment of chronic wounds, including indications for systemic antibiotics, require refinement of the diagnostic process that leads to their use in the first place. The treatment employed therein is also susceptible to improvement. A shift towards favoring local bacterial management and away from the regular use of systemic antibiotic therapies as the main approach to encourage healing can be most effective. In our practice, this has been made possible through FL-imaging, which allows us to visually determine high bacterial presence with a sensitivity 4 times greater than that of clinical signs and symptoms alone [8] (PPV 93–100% [40,41]). There is substantial evidence validating this technology’s high diagnostic accuracy and ability to detect most bacterial pathogens [20], whether in planktonic or in a biofilm state [19,42].

Our preliminary data shows that 70% of the cases in which this fluorescence imaging technology was used resulted in a therapeutic change, and only in 4% were systemic antibiotics prescribed. The impact of this approach on wound healing at our site is yet to be determined as we continue to follow our cohort prospectively. However, a recent RCT by Rahma et al. showed a 2-fold increase (22 vs. 45%) in 12-week healing rates on those DFUs whose therapeutic conduct had been influenced by FL-images. DFU healing rates more than doubled in the study arm while the antibiotic prescription rate was kept under 10% [17]. Price et al. documented an increase in healing rates of outpatient DFUs by 23% while at the same time decreasing systemic antibiotic consumption by 33% after incorporating FL-imaging in their practice [16]. Another study by Cole et al. quantified an average reduction in wound area of 27.7% per week after the implementation of targeted debridement guided by FL-imaging [43].

We believe the results obtained by these authors speak to how FL-imaging is visually and objectively showing the importance of a markedly localized and topical management of bacterial loads and biofilm. Biofilm in chronic wounds conveys bacterial protection from antibiotics, such that antibiotics alone have little to no effect in biofilm disruption, and ultimately, bacterial eradication. Bacteria living in a biofilm community are not only capable of protecting themselves but other species living within the same community, as well as future bacterial generations from antibiotics. It is now recognized that antibiotic-resistance genes can be transmitted to future generations [44]. So, addressing biofilm today may impact the antibiotic efficacy of tomorrow [1,44,45]. With the weight of this evidence in mind, we concluded that optimizing outcomes for the patients we treat would start by changing the wound care paradigm itself, that as an added benefit could impact positively antibiotic consumption and resistance. A combination of disruptive, biofilm-targeted, and repetitive measures has been implemented by us with the aid of the FL images. This approach is necessary to change the course of the wound once biofilm has been established [3], and to ensure the effect of topical antibiotics. Based on our findings, systemic antibiotics were rarely needed, we theorize that the enhancement of local treatment has deemed it obsolete in a great number of cases. Systemic antibiotics can then be considered an adjuvant therapy for those cases where an overlaying acute infection has occurred [6,44] or complications have ensued [34].

**Figure 2:**
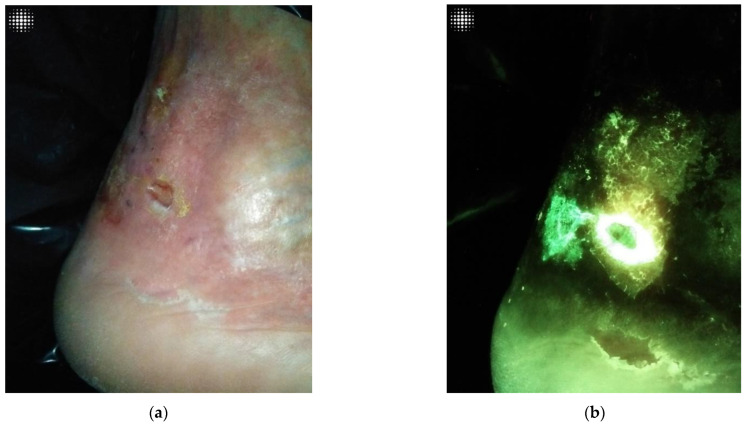
(**a**). Harmless wound on clinical inspection. (**b**) The same wound assessed under FL-imaging, demonstrating cyan positive signal, indicative of *Pseudomonas*. This was later confirmed by microbiology.

**Figure 3:**
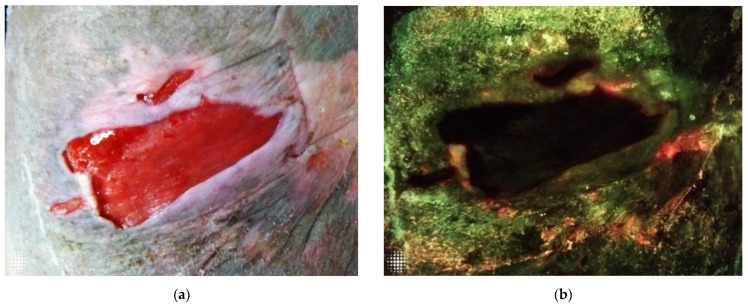
(**a**). Standard image of a wound without any obvious clinical signs and symptoms of infection on physical examination. (**b**) The same wound assessed under FL-imaging, demonstrating a red positive signal, indicative of high bacterial loads in the peri-wound area.

The implementation of FL- imaging in our clinical practice has clarified for us many of the ambivalences that exist in wound care, particularly in wound assessment and the clinical conduct derived from it. Figure 1a,b, Figure 2a,b, and Figure 3a,b illustrate how a wound that looks innocuous on physical examination can be harbouring high bacterial loads containing harmful microbes. In agreement with some of the authors before us, we have come to realize the need for repeated and more aggressive debridement [44]. This supports the notion that aggressive measures are the only way forward to prevent or disturb the formation of biofilm, which is essential to unblock the path toward wound healing and allow antimicrobials to do their job. Point-of-care imaging has brought to light the need for a workflow that is in keeping with antibiotic stewardship efforts.

We emulated a workflow from a Delphi consensus published by Oropallo et al. [18] that was logical to follow and easily understood, not only by our staff, but by the patients themselves. Increased patient involvement was another exciting result, as the ability to show patients a visual depiction of the status of their wounds facilitated their understanding on the importance of wound care. Further, this approach was observed to reduce patient requests of “needing” to be prescribed antibiotics; such patient requests were highlighted by Lipsky et al. [10] as one of the underlying factors in wound care antibiotic misuse.

## 5. Recommendations for Future Research

Ongoing data collection at our outpatient wound clinic will aim to describe in further detail the impact of incorporating this new technology into our day-to-day workflow. We will aim to evaluate this impact through several aspects. We will further evaluate how clinical decision-making changed after this technology was incorporated during the assessment of wounds. This will be achieved by a pre and post-interventional analysis of prescription rates, rates of debridement, the extension of debridement, and other wound hygiene aspects. Future research could aim to determine how these changes impact the healing rates of chronic wounds and the time to heal.

## 6. Clinical Implications for Health Managers and Policymakers

The urgency of further developing and adhering to practical strategies to overcome the growing threat of antibiotic resistance cannot be overstated. Practitioners, hospitals, and policymakers alike should partner in searching for the most cost-effective and applicable strategies to do so. Changes begin by modifying our status quo by identifying the areas susceptible for improvement in our day-to-day practices. We have found in Fluorescence imaging a valuable addition to our practice. This device promotes and enables accurate physical removal of bacteria allowing for a proactive and comprehensive treatment approach. All these characteristics have great potential to translate into less antibiotic use overall, as demonstrated in previous studies cited herein. We believe guidelines should continue to strengthen their support towards incorporating this technology as part of the usual clinical workflow. Further, we propose it has the potential to be a part of the standard of care for chronic wound patients.

## 7. Conclusions

We report that global outpatient chronic wound antibiotic prescribing rates are alarmingly high. Chronic wound patients, particularly those that have multiple comorbidities and are of advanced age and thus at risk of complications from infections, deserve priority in our efforts to improve their treatment, and this includes systemic antibiotic prescribing practices. Interventions to heighten awareness and disclosure of prescribing trends (such as national registries) have had a positive impact on lowering prescribing rates in multiple countries. Several groups have reported on a new strategy at the point of care, that are translating into more appropriate antibiotic prescribing after FL-imaging of bacterial burden during clinical inspection. The preliminary results reported herein are in line with antibiotic prescribing reduction obtained by other authors with the implementation of this technology with the added, important benefit of improving healing rates simultaneously.

We attribute this decrease in prescribing after incorporation of FL-imaging to four key elements: (1) earlier and localized detection of the bacteria at a stage where it could be proactively managed through hygiene and topical antimicrobial strategies, (2) more aggressive hygiene approaches in regions the images demonstrated had bacterial burden, and (3) willingness to switch the treatment plan as soon as it was deemed ineffective. (4) A new ability to verify the effectiveness of the debridement or cleansing session immediately after it was performed, thus allowing opportunity for correction.

## Figures and Tables

**Table 1:** Reported systemic antibiotic prescribing rates in outpatient wound care.

Reference	Country	Wound Types *	% of Patients Prescribed Antibiotics	Intervention Implemented	% of Patients Prescribed Antibiotics after Intervention	Notes on Prescribing
Serena et al. (2021) [11]	USA	DFU PU VLU SSI Others	25.7%	Moleculight^®^ fluorescence imaging of bacterial location and load	-	Prescription rate was taken at time of enrolment in the single time-point study and was based on standard of care (pre-intervention); postinterventional rate was not recorded
Price (2020) [16]	UK	DFU	67%	Moleculight^®^ fluorescence imaging of bacterial location and load	22.1% **	Healing rates improved by 23% at 12 weeks (from 38% to 49%)
Gürgen (2014) [7]	Norway	DFU VLU AU A-VU PU unspecified	53.3%	-	-	Prospective observational study of 105 patients, who had received at least one course of systemic antibiotics at any point during care for their wound
Öien et al. (2013) [24]	Sweden	DFU	71%	National quality assessment registry for ulcer care	29% *p* = 0.0001	Median healing time decreased by 83 days in 3 years (adjusted for ulcer size)
		VLU				
		AU				
		A-VU PU unspecified				
Howell-Jones et al. (2006) [22]	UK	DFU VLU AU A-VU PU	68.3%	-	-	% of patients who received at least one course of systemic antibiotics during the duration of the wound, compared to 29.3% in non-wound patients (*p* < 0.0001)
Tammelin et al. (1998) [23]	Sweden	PU Foot and leg ulcers Unspecified ulcers	60.1%	-	-	% of patients (n = 665) that had received at least one course of systemic antibiotics in the last 6 months for their wound

* DFU: Diabetic Foot Ulcer, VLU: Venous Leg Ulcer, AU: Arterial Ulcer, VU: Venous Ulcer, A-VU: Arterial Venous Ulcer, PU: Pressure Ulcer/Injury. ** Systemic antibiotics prescribed at any point during care for their wound.

## Data Availability

As this was a retrospective analysis of our clinical practice, data is not publicly available. Literature review was performed in the Pubmed and Cochrane databases, and all references are appropriately cited.

## References

[1] Bowler P.G., Duerden B.I., Armstrong D.G. (2001). Wound Microbiology and Associated Approaches to Wound Management. Clin. Microbiol. Rev..

[2] CDC (2016). CDC: 1 in 3 Antibiotic Prescriptions Unnecessary. https://www.cdc.gov/media/releases/2016/p0503-unnecessary-prescriptions.html.

[3] Attinger C., Wolcott R. (2012). Clinically Addressing Biofilm in Chronic Wounds. Adv. Wound Care.

[4] Ovington L. (2003). Bacterial toxins and wound healing. Ostomy Wound Manag..

[5] Rahim K., Saleha S., Zhu X., Huo L., Basit A., Franco O.L. (2017). Bacterial Contribution in Chronicity of Wounds. Microb. Ecol..

[6] Bowler P.G. (2002). Wound pathophysiology, infection and therapeutic options. Ann. Med..

[7] Gürgen M. (2014). Excess use of antibiotics in patients with non-healing ulcers. EWMA J..

[8] Le L., Baer M., Briggs P., Bullock N., Cole W., DiMarco D., Hamil R., Harrell K., Kasper M., Li W. (2021). Diagnostic Accuracy of Point-of-Care Fluorescence Imaging for the Detection of Bacterial Burden in Wounds: Results from the 350-Patient Fluorescence Imaging Assessment and Guidance Trial. Adv. Wound Care.

[9] Juneja D., Nasa P., Singh O. (2012). Severe sepsis and septic shock in the elderly: An overview. World J. Crit. Care Med..

[10] Lipsky B.A., Dryden M., Gottrup F., Nathwani D., Seaton R.A., Stryja J. (2016). Antimicrobial stewardship in wound care: A Position Paper from the British Society for Antimicrobial Chemotherapy and European Wound Management Association. J. Antimicrob. Chemother..

[11] Serena T.E., Gould L., Ousey K., Kirsner R.S. (2021). Reliance on Clinical Signs and Symptoms Assessment Leads to Misuse of Antimicrobials: *Post hoc* Analysis of 350 Chronic Wounds. Adv. Wound Care.

[12] Howell-Jones R.S., Wilson M.J., Hill K.E., Howard A.J., Price P.E., Thomas D. (2005). A review of the microbiology, antibiotic usage and resistance in chronic skin wounds. J. Antimicrob. Chemother..

[13] Nicolle L. (2014). Antimicrobial stewardship in long term care facilities: What is effective?. Antimicrob. Resist. Infect. Control..

[14] Dryden M., Johnson A.P., Ashiru-Oredope D., Sharland M. (2011). Using antibiotics responsibly: Right drug, right time, right dose, right duration. J. Antimicrob. Chemother..

[15] Caldwell M.D. (2020). Bacteria and Antibiotics in Wound Healing. Surg. Clin. N. Am..

[16] Price N. (2020). Routine Fluorescence Imaging to Detect Wound Bacteria Reduces Antibiotic Use and Antimicrobial Dressing Expenditure While Improving Healing Rates: Retrospective Analysis of 229 Foot Ulcers. Diagnostics.

[17] Rahma S., Woods J., Brown S., Nixon J., Russell D. (2022). The Use of Point-of-Care Bacterial Autofluorescence Imaging in the Management of Diabetic Foot Ulcers: A Pilot Randomized Controlled Trial. Diabetes Care.

[18] Oropallo A.R., Andersen C., Abdo R., Hurlow J., Kelso M., Melin M., Serena T.E. (2021). Guidelines for Point-of-Care Fluorescence Imaging for Detection ofWound Bacterial Burden Based on Delphi Consensus. Diagnostics.

[19] Jones L.M., Dunham D., Rennie M.Y., Kirman J., Lopez A.J., Keim K.C., Little W., Gomez A., Bourke J., Ng H. (2020). In vitro detection of porphyrin-producing wound bacteria with real-time fluorescence imaging. Futur. Microbiol..

[20] Raizman R., Little W., Smith A.C. (2021). Rapid Diagnosis of Pseudomonas aeruginosa in Wounds with Point-of-Care Fluorescence Imaging. Diagnostics.

[21] Okeahialam N.A., Thakar R., Sultan A.H. (2022). The clinical progression and wound healing rate of dehisced perineal tears healing by secondary intention: A prospective observational study. Eur. J. Obstet. Gynecol. Reprod. Biol..

[22] Howell-Jones R.S., Price P.E., Howard A.J., Thomas D.W. (2006). Antibiotic prescribing for chronic skin wounds in primary care. Wound Repair Regen..

[23] Tammelin A., Lindholm C., Hambraeus A. (1998). Chronic ulcers and antibiotic treatment. J. Wound Care.

[24] Öien R.F., Forssell H.W. (2013). Ulcer healing time and antibiotic treatment before and after the introduction of the Registry of Ulcer Treatment: An improvement project in a national quality registry in Sweden. BMJ Open.

[25] Miller L.G., Eisenberg D.F., Liu H., Chang C.-L., Wang Y., Luthra R., Wallace A.E., Fang C., Singer J., Suaya J.A. (2015). Incidence of skin and soft tissue infections in ambulatory and inpatient settings, 2005–2010. BMC Infect. Dis..

[26] Hurley H.J., Knepper B.C., Price C.S., Mehler P.S., Burman W.J., Jenkins T.C. (2013). Avoidable Antibiotic Exposure for Uncomplicated Skin and Soft Tissue Infections in the Ambulatory Care Setting. Am. J. Med..

[27] WHO (2017). The World Is Running Out of Antibiotics, WHO Report Confirms.

[28] Dowd S.E., Delton Hanson J., Rees E., Wolcott R.D., Zischau A.M., Sun Y., White J., Smith D.M., Kennedy J., Jones C.E. (2011). Survey of fungi and yeast in polymicrobial infections in chronic wounds. J. Wound Care.

[29] Dowd S.E., Sun Y., Secor P.R., Rhoads D.D., Wolcott B.M., James G.A., Wolcott R.D. (2008). Survey of bacterial diversity in chronic wounds using Pyrosequencing, DGGE, and full ribosome shotgun sequencing. BMC Microbiol..

[30] Gjødsbøl K., Christensen J.J., Karlsmark T., Jørgensen B., Klein B.M., Krogfelt K.A. (2006). Multiple bacterial species reside in chronic wounds: A longitudinal study. Int. Wound J..

[31] Bjarnsholt T., Kirketerp-Møller K., Jensen P., Madsen K.G., Phipps R.K., Krogfelt K.A., Høiby N., Givskov M. (2008). Why chronic wounds will not heal: A novel hypothesis. Wound Repair Regen..

[32] van Buul L.V., van der Steen J.T., Veenhuizen R.B., Achterberg W.P., Schellevis F.G., Essink R.T., Hertogh C.M. (2012). Antibiotic use and resistance in long term care facilities. J. Am. Med. Dir. Assoc..

[33] Rhee S.M., Stone N.D. (2014). Antimicrobial stewardship in long-term care facilities. Infect. Dis. Clin. N. Am..

[34] Wounds International (2016). IWII: Wound Infection in Clinical Practice. https://www.woundsinternational.com/resources/details/iwii-wound-infection-clinical-practice.

[35] Wounds International (2016). IWII Wound Infection in Clinical Practice, International Consensus Document. https://www.woundsme.com/uploads/resources/9b549b9d8a74b2c69a7773aa13157376.pdf.

[36] Yogo N., Gahm G., Knepper B.C., Burman W.J., Mehler P.S., Jenkins T.C. (2016). Clinical Characteristics, Diagnostic Evaluation, and Antibiotic Prescribing Patterns for Skin Infections in Nursing Homes. Front. Med..

[37] O’Meara S. (2013). Antibiotics and antiseptics for venous leg ulcers (Review). Cochrane Database Syst. Rev..

[38] Öien R.F., Weller W.C. (2014). The Swedish national quality Registry of Ulcer Treatment (RUT): How can ‘RUT’ inform outcome measurement for people diagnosed with venous leg ulcers in Australia?. Wound Pract. Res..

[39] Gottrup F., Apelqvist J., Bjansholt T., Cooper R., Moore Z., Peters E.J.G., Probst S. (2013). EWMA Document: Antimicrobials and Non-healing Wounds Evidence, controversies and suggestions. J. Wound Care.

[40] Raizman R., Dunham D., Lindvere-Teene L., Jones L.M., Tapang K., Linden R., Rennie M.Y. (2019). Use of a bacterial fluorescence imaging device: Wound measurement, bacterial detection and targeted debridement. J. Wound Care.

[41] Rennie M.Y., Dunham D., Lindvere-Teene L., Raizman R., Hill R., Linden R. (2019). Understanding Real-Time Fluorescence Signals from Bacteria and Wound Tissues Observed with the MolecuLight i:X(TM). Diagnostics.

[42] Lopez A.J., Jones L.M., Reynolds L., Diaz R.C., George I.K., Little W., Fleming D., D’Souza A., Rennie M.Y., Rumbaugh K.P. (2021). Detection of bacterial fluorescence from in vivo wound biofilms using a point-of-care fluorescence imaging device. Int. Wound J..

[43] Cole W., Coe S. (2020). Use of a bacterial fluorescence imaging system to target wound debridement and accelerate healing: A pilot study. J. Wound Care.

[44] Bowler P., Murphy C., Wolcott R. (2020). Biofilm exacerbates antibiotic resistance: Is this a current oversight in antimicrobial stewardship?. BMC.

[45] Bowler P.G. (2018). Antibiotic resistance and biofilm tolerance: A combined threat in the treatment of chronic infections. J. Wound Care.

